# Inefficiency, heterogeneity and spillover effects in maternal care in India: a spatial stochastic frontier analysis

**DOI:** 10.1186/s12913-015-0763-x

**Published:** 2015-03-25

**Authors:** Yohannes Kinfu, Monika Sawhney

**Affiliations:** Centre for Research and Action in Public Health, University of Canberra, University Drive, ACT 2602 Bruce, Australia; Bachelor of Public Health Program, College of Health Professions, One John Marshall Drive, Prichard Hall, Room number 309, Huntington, WV 25755 USA

**Keywords:** Institutional delivery, Efficiency of institutional delivery in India, Health efficiency analysis, Stochastic frontier model, Spatial stochastic frontier analysis

## Abstract

**Background:**

Institutional delivery is one of the key and proven strategies to reduce maternal deaths. Since the 1990s, the government of India has made substantial investment on maternal care to reduce the huge burden of maternal deaths in the country. However, despite the effort access to institutional delivery in India remains below the global average. In addition, even in places where health investments have been comparable, inter- and intra-state difference in access to maternal care services remain wide and substantial. This raises a fundamental question on whether the sub-national units themselves differ in terms of the efficiency with which they use available resources, and if so, why?

**Methods:**

Data obtained from round 3 of the country’s District Level Health and Facility Survey was analyzed to measure the level and determinants of inefficiency of institutional delivery in the country. Analysis was conducted using spatial stochastic frontier models that correct for heterogeneity and spatial interactions between sub-national units.

**Results:**

Inefficiency differences in maternal care services between and within states are substantial. The top one third of districts in the country has a mean efficiency score of 90 per cent or more, while the bottom 10 per cent of districts exhibit mean inefficiency score of as high as over 75 per cent or more. Overall mean inefficiency is about 30 per cent. The result also reveals the existence of both heterogeneity and spatial correlation in institutional delivery in the country.

**Conclusions:**

Given the high level of inefficiency in the system, further progress in improving coverage of institutional delivery in the country should focus both on improving the efficiency of resource utilization—especially where inefficiency levels are extremely high—and on bringing new resources in to the system. The additional investment should specifically focus on those parts of the country where coverage rates are still low but efficiency levels are already at a high level. In addition, given that inefficiency was also associated inversely with literacy and urbanization and positively related with proportion of households belonging to poor households, investment in these areas can also improve coverage of institutional delivery in the country.

## Background

Maternal mortality—the death of women during pregnancy, childbirth, or in the 42 days after delivery—remains one of the greatest public health challenges of our time.The Fifth Goal of the United Nations Millennium Declaration (MDG 5) of 2000 calls for a reduction in maternal mortality ratio (MMR) in all countries so that by 2015 it is one quarter of its 1990 level [1]. However, the progress recorded so far has been relatively slow so much so that maternal mortality is often described as the most seriously “off track” of all the health-related MDGs [2-5].

Recent estimates show that, globally, more than a-quarter-of a million women die each year because of childbirth and pregnancy complications [6]. Some 99 per cent of these deaths occur in the developing world and about half of these total come from just six countries—including India, Nigeria, Democratic Republic of Congo, Pakistan, Indonesia and Sudan—which make up not more than a quarter of the world population [6,7]. India alone accounts for 19% of the global total—the highest for any country in the world—with some two-third of which coming from just nine of its 35 states and federal territories [8].

Maternal death is a great tragedy because most of the deaths associated with pregnancy, childbirth, or in the 42 days after delivery are preventable through effective interventions, such as by promoting institutional delivery that ensures women access to skill birth attendants [9-14]. The National Population Policy of India stipulates a similar strategy to curb the high level of maternal mortality that prevails in the country [15]. The Child Survival and Safe Motherhood Program of 1992–1997 and the Phase-1 of the Reproductive and Child Health Program (RCH-1) implemented during 1997–2004 also constitute part of the same effort to improve maternal and newborn health in India [16,17]. Following the adoption of the MDGs, the Indian government further reinforced its efforts through introducing a system of conditional cash assistance to mothers as they attend delivery and post-delivery care. These interventions were particularly noteworthy given their emphasis on reaching rural communities and women belonging to lower socio-economic status in the country [18]. The country also instituted a district-based decentralized approach that ensures follow up and program ownership at the grass root level.

However, despite successive initiatives and efforts on the part of the government, inter-state and intra-state variations in institutional delivery remain wide in India [19]. The same is the case with respect to other critical inputs required for improving maternal health in the country. For instance, while over 70 per cent of health facilities in relatively well-resourced districts of the country had highly trained practitioners (such as lady-medical-officer, an obstetrician, or a gynecologist) in some districts located in less developed parts of the country this proportion was fewer than 2 per cent. Similarly, annual per capita public expenditure on health varies considerably across the different parts of the country [20]. What is even more concerning is the fact that the returns from past interventions also seem to be more uneven, with some districts achieving limited outcome than others even when they had comparable health inputs. This raises a fundamental question on whether the districts in the country differ in terms of the level of efficiency with which they use the resources available to them, and if so, why?

To address this question, the efficiency of institutional delivery was analyzed using a stochastic frontier approach. While there are a few previous studies on aspects of health care efficiency, the present paper introduces several novel dimensions [21-25]. First, the analysis covers the entire country, uses data that are more recent and focuses on districts, which constitute the basic unit of the country’s health system. Second, given the expectation that districts located in close proximity are able to interact with each other and influence each other’s output and efficiency levels, through competition and/or learning effects, spatial dependence and spillover effects have been explicitly introduced into the present analysis. This is the first time that such a model is introduced in the health care efficiency literature in any part of the world. Third, given India is a heterogeneous country with respect to level of development, governance and models of social service provision that could significantly influence and distort inefficiency estimates, the study also controls for heterogeneity in the analysis. Finally, to the best of our knowledge, the present paper is also the only study that has so far looked into inefficiencies in institutional delivery care in India. Hence, by doing so, the present study not only provides new evidence on India but also introduces alternative analytical dimensions that can be applied to other settings.

The remaining part of the article is organized as follows: The following section reviews the stochastic frontier model and introduces the methodology and data used in the study. The empirical results and discussion are presented next followed by some concluding remarks in the final part of the paper.

## Methods

### Classical stochastic health frontier Model

Generally, inefficiency analysis begins with estimation of a production/ cost frontier using either a deterministic or stochastic approach [26,27]. Because deterministic models do not take into account of the effects of random factors nor the factors beyond the control of the producer, and because the database in our disposal does not capture input prices to support estimation of a cost frontier function, this paper employs a production-based stochastic frontier model [28-30]. Typically, a standard stochastic health frontier model is written as follows [31-34]:

1 $$ {y}_i = \alpha + {x}_i\ \beta +{v}_i - {u}_i $$

$$ {\xi}_i = {v}_i - {u}_i; {v}_i \sim N\ \left(0;\ {\sigma_v}^2\right);\sim \eta $$

where, *y*_*i*_ is a scalar output of the ***i***^***th***^ productive unit (where _i = 1, ….n_); *x*_*i*_ represents a k X 1 vector of inputs and β is a k X 1 vector of unknown parameters to be estimated from the model. *ξ*_*i*_ is a composite error term, representing the sum (or the difference) of the disturbance term (*v*_*i*_)—representing the measurement and specification error—and the inefficiency component denoted by *u*_*i*_.

Estimation of a stochastic frontier model requires a range of assumptions on the error components. Primarily, the model assumes that both the symmetric error term (v) and the non-negative inefficiency component (u) are independent of each other and iid across observations [26,28]. In practice, v is also usually assumed to be normal N (0; σ_v_^2^) while the distribution of u (η) can be selected from half-normal [28,35], exponential [29], truncated normal [36], gamma [37] or log-normal [38] distributions.

In many real world applications, the classical stochastic health frontier model produces biased inefficiency estimates especially in settings where observational units are vastly different from each other [39], a case which can also be relevant to India where some parts of the country are more developed than others. Greene [31,39] addresses the bias in the classical model by introducing a heterogeneity variable either into the production function or in the inefficiency distribution of the original model. Hence, if *h*_*i*_ denote a variable measuring area-specific heterogeneity, a model with a heterogeneity component in the production function can easily be inferred from equation 1 as follows [31,39].

2 $$ \begin{array}{l}{y}_i=\alpha +{x}_i\beta +{h}_i\gamma + {v}_i - {u}_i\\ {}{u}_i=\theta +{z}_i\delta + \kern0.5em \widehat{u}\\ {}{v}_i \sim N\ \left(0;\ {\sigma_v}^2\right);\end{array} $$

Greene [31] characterizes such a model, which places the unmeasured heterogeneity in the production function as the true fixed effects model. This model essentially produces a neutral shift of the function, specific to each area. As specified in Greene [31], it is also possible to place the heterogeneity measure in the mean of the inefficiency component, as shown below:

3 $$ \begin{array}{l}{y}_i=\alpha +{x}_i\beta + {v}_i - {u}_i\\ {}{u}_i=\theta +{z}_i\delta + {h}_i\gamma + \widehat{u}\\ {}{v}_i \sim N\ \left(0;\ {\sigma_v}^2\right);\end{array} $$

Model (2) and (3) are essentially identical, and in moderately sized samples, these re-specifications represent a minor reformulation of the familiar classical stochastic frontier model [31].

While Greene’s re-specifications improve the performance of the classical frontier model by controlling for area level heterogeneities in the data, in many instances, particularly at a lower level of geography, contiguous geographic units also tend to interact with each other more directly which leads to various forms of spatial interdependence or interaction between health production units. For instance, in India, as in many other parts of the world, people cross boundaries to seek care and services in neighboring districts. Moreover, there is also a tendency among neighboring districts to compete for scares health resources (such as human resources for health or health budget from state authorities) or emulate each other’s way of doing things (in a good or bad way) as they go about in their day to day business of providing care to their respective population. Interactions such as these are non-trivial because they can potentially affect both the production function and the mean efficiency distribution of the familiar stochastic health frontier model. Besides, the correlation itself lends to violation of the conventional assumption of independence of observational units [40]. There is, therefore, both a theoretical as well as a statistical reason for us to look beyond the standard approach—which views the atomistic agent (in our case the district) as a decision maker acting in isolation—and capture the interactions between health production agents in the system more directly.

Methodologically, this requires us to use spatial models that are capable of identifying and measuring spillover effects (or spatial correlation) in the system. A number of studies in other disciplines [40-46] have applied such models, but the approach has not been previously developed and tested for sub-national health care inefficiency analysis despite the fact that spillover effects and spatial externalities seem to be quite a common place in national health systems around the globe.

### Stochastic health frontier model with spillover effects

Generally, spatial externalities (or spillover effects) can be hypothesized to manifest in the health sector in one of the following three ways. First, when the level of output in neighboring areas influences the output level in the geographic area of interest. Second, when the level of inputs in neighboring areas influences the output level of the geographic area of interest. Third, where mean efficiency levels of contiguous units are spatially correlated to each other. Thus, using equations (2) and (3) as a starting point and borrowing from the literature on spatial econometrics/Statistics [40-42,45,47,48], a general form of a spatial stochastic health frontier model can be formulated as follows:

4 $$ \begin{array}{l}y = \rho Wy+\beta X + \tau Wx + \xi \\ {}\xi = v-u\\ {}v = \lambda Wv+\widehat{v}\\ {}u = \varphi Wu+\delta Z + \omega Wz + \widehat{u}\end{array} $$

Where:

- y is an n x 1 vector of observations on the dependent variable (in this paper this represents observations on institutional delivery rate);
- X is an n x k matrix of observations on input variables that directly influence the production function, and *β* is the corresponding k x 1 parameter vector.
- Z is an n x k matrix of exogenous variables that affect inefficiency (but not the production frontier) and *δ* is the corresponding k x 1 parameter vector.
- *W* is an n x n spatial-weighting matrix (with 0 diagonal elements) usually specified in terms of first-order continuity relations or as functions of distance. In many applications, the weighting-matrix for all lag variables is assumed to be the same, but there is no methodological restriction to apply a different set, when and if required.
- *Wy*, *Wx*, *Wû*, and *Wz*,are n x 1 vectors representing spatial lags and *ρ*, *τ*, *φ* and *ω* are the corresponding scalar parameters measuring degrees of spatial interactions with respect to output, inputs, inefficiency level and the exogenous variables affecting inefficiency, respectively. Hence, *ρ*, for example, measures the degree to which access to level of institutional delivery in neighboring districts influence the level of service coverage in the district of interest.
- *Wv* are spatial errors (with a coefficient *λ*)

From the more general model in (3) a variety of special spatial health frontier models can be derived by imposing restrictions on any of the specified lag variables. For example, setting the lag values for x, z, u and v to zero produces a model with spatial dependence at the level of output:

5 $$ \begin{array}{l}y = \rho Wy+\beta X + v-u\\ {}u = \delta Z + \kern0.5em \widehat{u}\end{array} $$

Note that as before *v* represents measurement and specification error and is assumed to be normal N (0; σ_v_^2^), while the distribution of *u* (η) can be selected from half-normal [28,35], exponential [29], truncated normal [36], gamma [37] or log-normal [38] distributions.

Following the nomenclature in spatial statistics/econometrics [47-50], this model may be referred to as autoregressive-regressive spatial stochastic frontier model (ARRSF model, for short)). In the present case, such a model enables us to capture how institutional delivery rate in the district of interest depends not only on the inputs that the district itself puts into the system but also on the level of output achieved by neighboring areas. We note that if there is no spatial dependence (meaning y does not depend on neighboring y values) then *ρ* will be statistically indistinguishable from zero. On the other hand, a positive and significant value suggests the existence of spatial externality in the system. Model (4) can be further extended to allow for efficiency level from neighboring observations (created by *Wu*) to affect output level in the district of interest as shown in (5):

6 $$ \begin{array}{l}y = \varphi Wu+\beta X + v-u\\ {}u = \delta Z + \kern0.5em \widehat{u}\end{array} $$

Alternatively, Model (4) can be re-written as shown in (6) below to allow for one or more input variables from neighboring observations (created by Wx) to affect the output level in the district of interest:

7 $$ \begin{array}{l}y = \rho Wy+\beta X + \tau Wx+v-u\\ {}u=\delta Z + \kern0.5em \widehat{u}\end{array} $$

As noted earlier, the kx1 parameter vector *τ* measures the marginal impact of the input variables from neighboring observations on the dependent variable y. Hence, (7) can be thought of as a spatial stochastic frontier equivalent of what is generally known as a spatial Durban model [40,42,47]. Similarly, a model that extends the spatial dependency to the inefficiency distribution leads to the following:

8 $$ \begin{array}{l}y = \beta X + v-u\\ {}u = \varphi Wu+\delta Z + \kern0.5em \widehat{u}\end{array} $$

The reader will note that the heterogeneity components discussed by Greene [31,39] and described in (2) and (3) can be easily introduced into these spatial models. For example, a spatial stochastic frontier model that combines both spatial dependence (with respect to input, output and inefficiency components) as well as heterogeneity at the level of the production function can be expressed as follows:

9 $$ \begin{array}{l}y = \rho Wy+\beta X + \tau Wx + \gamma H+v-u\\ {}u = \varphi W\kern0.5em \widehat{u}+\delta Z + \omega Wz + \kern0.5em \widehat{u}\end{array} $$

Note that H is an (n x k) matrix of variables measuring cross-area heterogeneity while *γ* is its associated k x 1 parameter vector. In the same vain, following [51,52] a model where the heterogeneity resides in the location of the inefficiency distribution can be rewritten as:

10 $$ \begin{array}{l}y = \rho Wy+\beta X + \tau Wx+v-u\\ {}u = \varphi W\kern0.5em \widehat{u}+\delta Z + \omega Wz + \gamma H+\kern0.5em \widehat{u}\end{array} $$

Similarly, as shown by [53] allowing the variance of the idiosyncratic term to be hetroskedastic would give:

11 $$ \begin{array}{l}y = \rho Wy+\beta X + \tau Wx+v-u\\ {}u = \varphi W\kern0.5em \widehat{u}+\delta Z + \omega Wz + \kern0.5em \widehat{u}\\ {}v = \gamma H\kern0.5em  + \widehat{v}\end{array} $$

In this paper, we fit the classical stochastic frontier model described in equation (1) and compare the results with the outcome from equation (2) that corrects for heterogeneity as well as with results from equations (4) and (4), which capture the effects of output and efficiency lags, respectively. In addition, to assess the effects of heterogeneity and spatial correlation corrected models on efficiency estimates we also generate and compare efficiency scores for each district from each of these models. As is common with the standard practice [51,54], the scores generated in this fashion would allow us to examine how well each decision-making agent, represented by a district in our analysis, was performing its function compared to the maximum possible potential, given current resources at its disposal. A district is generally classified as inefficient, if it is observed to have a coverage rate below the maximum level that can be attained from a given set of inputs. Theoretically, inefficiency scores range from 0 (the most efficient) to 1 (the least efficient), with values in between representing a shortfall of observed output from maximum feasible output.

In our analysis, we chose to focus on districts, because they are responsible for the allocation and management of health inputs in their respective jurisdictions. This in turn means that their success or otherwise is a good reflection of the success or failure of the country’s health system or more specifically the progress toward meeting the target for MDG 5 for the country.

### Data and variable description

The proposed analysis requires five sets of information: data on the output of interest; on input variables that directly affect the production function; on exogenous variables that affect the inefficiency distribution (but not the production function); on a variable (or set of variables) that capture(s) spatial heterogeneity in the data and finally a spatial-weighting matrix. The spatial-weighting matrix is required to generate spatially lagged variables that we will use in measuring the spatial correlation and spillover effects in the system.

Output is measured using institutional delivery rate reported for each district. We focused on institutional delivery because improving maternal health through promoting access to institutional delivery is an integral part of India’s primary health care agenda. Second, institutional delivery itself is one of the key interventions known to have the greatest impact on improving maternal health in the developing world [1,11-14]. Finally yet importantly, institutional delivery is also one of the indicators used for monitoring progress towards MDG5, a goal to which India is also a signatory.

In measuring the inputs to the production process, three indicators were selected: namely, the density of health facilities per 1000 square kilometer, proportion of facilities that received ‘untied funding’ in previous financial year and proportion of facilities in a district that had highly trained practitioners (i.e. a general surgeon or obstetrician /gynecologist/lady-medical-officer). These variables represent some of the key inputs required for providing safe delivery care. To these, we have also added a composite variable called *service readiness index* to capture the effects of availability of selected basic amenities, such as communication equipment, operating theater and a labour room in the facilitates reported in each area. This index was considered useful because even if facilities are provided with the required human and financial resources (and are made available within accessible distance), they will still not be fully ready to provide services unless they are also equipped with amenities that are vital for their function [53].

Furthermore, three exogenous variables were added into the analyses to look more closely into the determinants of inefficiency in the country. These included literacy rate, urbanization and proportion of households in the lowest wealth quintile. It should be noted that these variables as such do not constitute direct inputs to the production of institutional delivery, but we assume that they are part of the environment within which districts had to maximize their outputs, and, therefore, can exert an influence on the performance of the country’s health system. For instance, districts with high illiteracy rate may face resistance in promoting institutional delivery because of cultural barrier, and, as a result, may need to divert resources away from service provision to health promotion and advocacy purposes. Similarly, in communities where over all living standard is low women may still be unable to access services (even if they are available) due to lack of transport and associated transactional costs. This means that the presence (or absence) of such contextual variables, could individually or collectively facilitate (or hinder) the efficiency with which districts can achieve their stated health goal, but on their own the exogenous variables do not affect output levels as such.

A dummy variable was also introduced into the analysis to control for the existence of heterogeneity in the data. Hence, we assigned a value of one for all districts belonging to the so-called ‘backward’ states of India and a value of zero for the remaining districts in the country. These states which included Assam, Bihar, Chhattisgarh, Jharkhand, Madhya Pradesh, Orissa, Rajasthan, Uttar Pradesh and Uttarakhand contribute about two-third of maternal deaths in the country. About 42 per cent of the districts in the data belonged to these states. Table 1 shows descriptive statistics for all variables used in the paper.

**Table 1:** **List of variables and corresponding descriptive statistics, India, 2007-08**

**Variable:**			**Descriptive statistics**	
**Description**	**Type**	**Code**	**Mean**	**Standard deviation**
Institutional delivery rate (%)	Output	Y	50.34	23.70
Number of facilities per 1000 sq km	Input	*x*_1_	0.016	0.023
Facilities having Medical Officer/Obstetrician/Gynecologist (%)	Input	*x*_2_	28.62	20.82
Facilities received untied funding in previous financial year (%)	Input	*x*_3_	74.87	22.54
Facilities with selected basic amenities (%)	Input	*x*_4_	35.01	17.03
Population in lowest wealth quintile (%)	Exogenous	*z*_1_	18.92	17.33
Population residing in urban areas (%)	Exogenous	*z*_2_	25.25	17.55
Population literate age 7+ years (%)	Exogenous	*z*_3_	70.63	10.56
Districts in ‘backward state’ (%)	Heterogeneity	*H*_1_	41.58	49.33
Number of districts covered*				499
Contiguity-matrix used for creating lag variables**				
Total links (number)				3222
Minimum links (number)				0
Average links (number)				5.42
Maximum links (number)				10

Note: *Total number differs from those reported in DHLS – 3 because ours is restricted only to districts with complete information on all variables needed for the analysis in the paper. See the Chorpleth map in Figures 1 and 2 for districts with missing data. **The matrix was based on publically available ‘shape (or co-ordinates) file containing the polygon information for each district in the country.

Moreover, we constructed two separate spatial lag variables for output and efficiency level of districts. Spatial lags are a weighted average of the values of the variables of interest for neighboring areas with the weight determined based on some measure of connectivity. In the present case, a contiguity-based weighting scheme, which involve assigning a weight of one for contiguous areas and a value of zero for non-contiguous districts, was applied [40,45,46,55]. This is one of the most common approaches for generating spatial lag variables [40,55]. Keeping with the practice, we also used identical spatial weighting matrix for both of our lag variables [45,46].

As shown in Table 1, the weighting-matrix produced over 3000 links (or contiguous units which are also known as neighbors), with each district having, on average, about five neighbors while some districts sharing border with as many as 10. The spatial data needed for creating the contiguity-matrix was obtained from open source shape files (or ‘co-ordinate files) for India. On the other hand, the remaining data used in the paper were extracted from India’s latest round district level household and facility survey (DLHS-3, 2007–08) [19]. These data were normalised around their respective means and transformed to a log-scale to control for the effects of different units of measurement. Analyses of data were performed using version 13 of the STATA software.

## Results and discussion

Table 2 presents maximum likelihood results for four variants of a log-linear Cob-Douglas stochastic production frontier model (see [56-65] for the log likelihood functions and maximum likelihood estimations). Model 1 shows the parameter values for the classical stochastic frontier model. Model 2 is for heterogeneity controlled model estimated using equation (2) discussed in Section “Methods”. Models 3 and 4 are based on equations (5) and (6), and capture the effects of output and efficiency lags on the output level of the district of interest, respectively. Following [66], for each of our four models we estimated both the production function and the inefficiency component in a single stage. The models reported in Table 2 were also fitted sequentially, starting with the standard stochastic frontier model, followed by the model with a heterogeneity element and finally by the two spatial stochastic frontier models that capture spillover effects in the data. Sequential modelling ensures that the models are nested and can be compared using standard statistical test.

**Table 2:** **Maximum likelihood estimates of classical and spatial stochastic frontier models, 2007–08, India**

	**MODEL 1**	**MODEL 2**	**MODEL 3**	**MODEL 4**
***Production function***				
Constant	0.8616 (0.0705)***	1.2178 (0.0652)***	1.7013 (0.0799)***	1.6790 (0.0794)***
x_1_	0.0822 (0.0282)***	0.0481 (0.0237)**	0.0746 (0.0238)***	0.0804 (0.0244)***
*x*_2_	0.1080 (0.0346)***	0.1532 (0.0281)***	0.1044 (0.0268)***	0.1201 (0.0266)***
x_3_	0.1587 (0.0378)***	0.0426 (0.0343)	−0.0207 (0.0344)	−0.0180 (0.0343)
x_4_	0.1557 (0.0351)***	0.0651 (0.0292)**	0.0814 (0.0274) ***	0.0857 (0.0280) ***
*γ* (Heterogeneity index)		−0.8317 (0.0566)***	−0.6972 (0.0573)***	−0.8623 (0.0559)***
*ρ* (Lagged output)			0.4588 (0.0493) ***	
*φ* (Inefficiency lag)				0.4465 (0.0462) ***
***Inefficiency***				
Constant	−0.2091 (0.2024)	−0.2193 (0.1780)	−0.7763 (0.2226)***	−0.9486 (0.2570)***
z_1_	0.6349 (0.1175)***	0.4457 (0.1014)***	0.3972 (0.1087)***	0.3827 (0.1134)***
z_2_	−0.6651 (0.1432)***	−0.6417 (0.1236)***	−0.9082 (0.1868)***	−0.9945 (0.2186)***
z_3_	−0.0874 (0.1114)	−0.0305 (0.1011)	−0.1132 (0.1123)	−0.1043 (0.1170)
Predicted mean inefficiency	0.4709	0.4592	0.3950	0.3756
Distributions of *u* and *v*				
δ_u_	1.0664	0.9989	0.7995	0.7437
δ_v_	0.4536	0.3088	0.3626	0.3852
λ	2.35	3.24	2.2049	1.9307
Log likelihood	−549.2938***	−468.0001***	−426.2494***	−423.7609***
N	499	499	499	499

Notes: Estimated standard errors in parenthesis. *** Indicates statistical significance at 99% level and ** indicates significance at 95% level.

Results for the classical model indicate that the coefficients for inputs not only have the correct sign but also that they are all statistically significant meaning that ‘untied funding’, more health facilities per square kilometres and more qualified health workers per facilities as well as facilities that are well equipped with basic amenities (such as labour room, operation theatre and communication equipment) will lead to significantly high rate of institutional deliveries in a district. These results remain consistent across the remaining three models except for ‘untied funding’ which tended to loose significance once heterogeneity and spatial interactions were controlled for probably because existing rules governing untied funding favours marginalised parts of the country. The magnitudes of the coefficients for the other variables also decline as we progressively introduce heterogeneity and spatial correlations into the analysis.

In all the four models, the asymmetry parameter, **λ,** is well above unity, which is an indication of inefficiency in the provision of maternal care service in the country. The predicted mean inefficiency from the classical stochastic frontier model (Model 1) is around 47 per cent but once heterogeneity and spatial interactions are incorporated both the mean inefficiency score and the estimated underlying standard deviation of u (δ_u_) change significantly. These are consistent with Greene’s [31,39] conjecture that unaccounted for heterogeneity was indeed showing up as inefficiency in the original model. The statistically significant coefficient for the heterogeneity indicator variable as well as the log-likelihood values for Model 1 and Model 2 also confirms that the model with a heterogeneity component handles the data notably better.

However, as discussed earlier Model 2 only corrects for heterogeneity and does not address the potential effects of spatial interactions between neighbouring districts on their respective output and efficiency level. As can be seen from the log-likelihood values for Model 3 and Model 4, correcting for spatial correlations significantly improve the model fit. Similarly, once spatial dependence in output and efficiency are controlled for the estimated underlying distribution of u (δ_u_) falls almost by 25 per cent (i.e. from 1 to between 0.75 and 0.8) and the predicted mean inefficiency almost by 20 per cent (i.e. from 0.48 to between 0.38 and 0.40). In both cases, the observed changes in the efficiency function were not accompanied by significant changes in the residual distribution (δ_v_) of the two models. This, in turn, reinforces our belief that the spatial stochastic frontier models add something important to specifying the inefficiency distribution of institutional delivery, beyond what we would expect from a heterogeneity corrected frontier model.

Further evidence on the existence of spill over effects comes from the coefficient for the lagged output variable (*ρ*). This estimate is both large and positive and highly statistically significant by standard criteria. This provides support for the conjecture that the coverage of institutional delivery in a district covaries with the level observed for its geographical neighbours. The corresponding lag coefficient for inefficiency (*φ*), which is equally large and highly significant, also shows clearly the impact of efficiency levels of neighbouring districts on the output of the districts of interest in the country.

Once the relative robustness of the spatially corrected models was verified at the aggregate level, we also examined the exact effect of such interactions on the efficiency level of each district in the country. This is important because both the magnitude and direction of the effect can hardly be expected to be uniform across all districts. Thus, we specifically, analysed the differences in efficiency estimates between models with and without spatial interactions, calculating the following measures of distance.

$$ {d}_i=\frac{Eff\ \left[ model\ 3,i\right]- Eff\ \left[ model2,i\right]}{Eff\ \left[ model\ 3,i\right]}\ x\ 100,\ \forall i=1, \dots \dots ..\ 499\ \left(for\  output\  lag\  corrected\  model\right) $$

$$ {d}_i=\frac{Eff\ \left[ model\ 4,i\right]- Eff\ \left[ model2,i\right]}{Eff\ \left[ model\ 4,i\right]}\ x\ 100,\ \forall i=1, \dots \dots ..\ 499\ \left(for\  efficiency\  lag\  corrected\  model\right) $$

Note that the efficiency estimate from model 2 represents efficiency estimates corrected only for heterogeneity but not for spatial correlations, while model 3 and for 4 are corrected for both heterogeneity and for output and efficiency correlations, respectively. Also note that the higher the value of the distance measure (*d*_*i*_*)* the greater the impact of neighbours on each other’s levels of efficiency, while its sign shows whether the interdependencies between districts are positive or not. Table 3 presents a summary of these estimates for the country.

**Table 3:** **Impact of spatial interactions on efficiency levels of districts, 2007–08, India**

**Magnitude of effect on efficiency level**	**Model 3**		**Model 4**	
	**[Output lag corrected]**		**[Efficiency lag corrected]**	
	**Number of districts affected**	**%**	**Number of districts affected**	**%**
Negative impact on efficiency	96	19.2	69	13.8
Efficiency increase of up to 9.99%	157	31.5	149	29.9
Efficiency increase between 10.0 – 29.99%	161	32.3	171	34.3
Efficiency increase of 30% or more	85	17.0	110	22.0
Total	499	100.0	499	100.0

**Figure 1:**
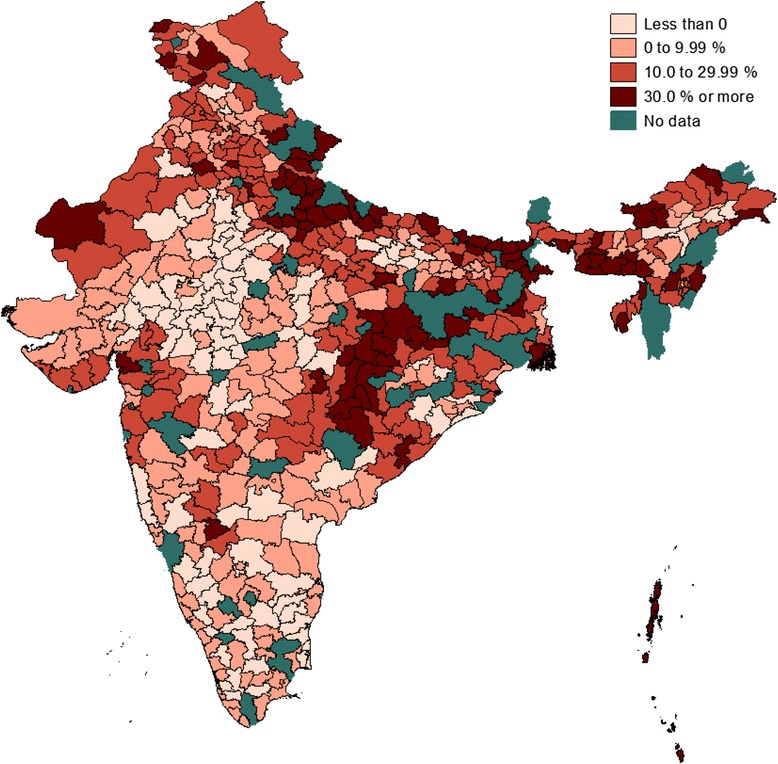
**Gains or loss in efficiency (%) due to output interaction.** Note: Score of less than zero implies efficiency loss and a positive value suggests efficiency gain.

**Figure 2:**
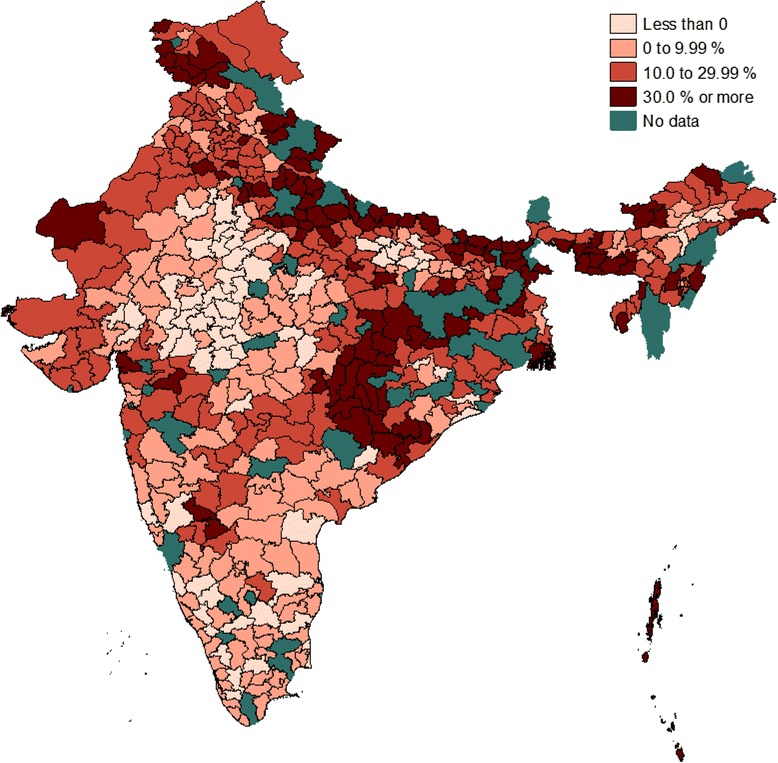
**Gains or loss in efficiency (%) due to efficiency interaction.** Note: Score of less than zero implies efficiency loss and a positive value suggests efficiency gain.

As can be seen from the table, spatial interaction is quite common in India’s health system, although the strength as well as the direction of the interactions seem to vary widely in the country. Results from model 3 which are corrected for output level interactions show that in about 19 per cent of the districts efficiency levels were adversely affected by the level of institutional delivery rates attained by their immediate neighbours. On the other hand, in one out of five districts the efficiency level was boasted by as high as 30 per cent due to the positive effect of the efficiency level attained by their neighbours. The estimates for the efficiency interaction model are slightly different but overall they point to the same direction. Detailed district-specific estimates on the magnitude of the effects of output and efficiency interactions on the efficiency level of each of the districts in the country are presented in Figures 1 and 2, respectively.

Table 4 presents mean inefficiency score summary for India’s state and territories obtained using Model 4, which is corrected for both heterogeneity and spatial dependence in the data. Shown on the same table are results from the model corrected only for heterogeneity. Both models clearly confirm the existence of huge difference in level of inefficiency between and within states. State level mean inefficiency scores corrected for heterogeneity and spatial dependence range from ten per cent or less in Poduchery, Goa and Daman and Diu states to over 50 per cent in Manipur, Jarkhand, Uttar Pradesh, Bihar, Meghalaya and Chhattisgarh states. In Bihar, 30 of the 34 districts covered in the analysis are operating below 90% of their maximum potential; while in Kerella 13 of the reported 14 districts have efficiency score of 75% or more. Overall, about two-third of the districts in the country had mean efficiency level of 75 per cent or less while the national mean efficiency score was about 60 per cent. Note that although the results from the model corrected only for heterogeneity broadly suggest similar a picture, in some cases the ranking and mean efficiency estimates differ significantly.

**Table 4:** **Inefficiency distribution by state and federal territories, 2007–08, India**

**State/Territory**	**INEFFICIENCY DISTRIBUTION for FINAL MODEL [MODEL 4 [i.e. Corrected for heterogeneity and spatial correlation for efficiency]**							**Corrected ONLY FOR heterogeneity: [Model 2]**		**Gains or loss in efficiency due to spatial interaction (%)**
	**Number of districts with inefficiency level:**				**Total**	**Mean inefficiency score**	**Rank**	**Mean inefficiency score**	**Rank**	
	**<10%**	**> = 10% and less than 25%**	**> = 25% and less than 70%**	**> = 70%**						
Daman & Diu	1	0	0	0	1	0.0713	1	0.2027	6	184.4
Goa	1	1	0	0	2	0.0929	2	0.1605	3	72.8
Puducherry	1	1	0	0	2	0.1067	3	0.1492	2	39.8
Kerala	4	9	1	0	14	0.1360	4	0.1390	1	2.2
Tamil Nadu	2	18	3	0	23	0.1631	5	0.1772	5	8.7
Lakshadweep	0	1	0	0	1	0.1661	6	0.1661	4	0.0
Andhra Pradesh	1	11	10	0	22	0.2499	7	0.3110	8	24.5
Gujarat	2	7	14	0	23	0.2749	8	0.3554	10	29.2
Tripura	0	2	2	0	4	0.2767	9	0.4712	16	70.3
Madhya Pradesh	2	20	20	1	43	0.2894	10	0.3014	7	4.2
Punjab	0	3	14	0	17	0.3120	11	0.4211	14	35.0
Uttaranchal	0	2	6	0	8	0.3164	12	0.5291	21	67.2
Karnataka	0	8	15	1	24	0.3174	13	0.3584	11	12.9
West Bengal	0	6	8	0	14	0.3225	14	0.4947	18	53.4
Andaman & Nicobar	0	1	1	0	2	0.3288	15	0.3288	9	0.0
Rajasthan	0	9	22	1	32	0.3435	16	0.3735	12	8.7
Maharashtra	1	8	21	2	32	0.3440	17	0.4153	13	20.7
Haryana	0	5	14	0	19	0.3468	18	0.4975	19	43.4
Arunachal Pradesh	0	2	11	1	14	0.3876	19	0.5343	22	37.9
Assam	0	5	16	1	22	0.3884	20	0.4677	15	20.4
Jammu & Kashmir	1	4	5	3	13	0.4104	21	0.5482	23	33.6
Orissa	0	10	10	5	25	0.4135	22	0.4882	17	18.1
Himachal Pradesh	0	0	10	0	10	0.4582	23	0.5196	20	13.4
Manipur	0	2	4	2	8	0.5110	24	0.6363	26	24.5
Jharkhand	0	1	2	1	4	0.5110	25	0.8064	27	57.8
Uttar Pradesh	1	8	41	15	65	0.5182	26	0.6201	24	19.7
Bihar	0	4	22	8	34	0.5310	27	0.6333	25	19.3
Meghalaya	0	1	1	5	7	0.6692	28	0.8321	28	24.3
Chhattisgarh	0	0	8	6	14	0.6745	29	0.8386	29	24.3
Total	17	149	281	52	499	0.3756		0.4592		22.3

## Conclusion

Efficiency analysis provides several benefits to health providers, planners and policy makers alike. First, the resulting analyses help stakeholders in identifying geographic units that may be able to attain better outcome without increased allocation of resources. Second, the evidence from the analysis can also provide information on those exogenous factors whose presence (or absence) affects the performance of services and ultimately health outcomes in the country. The paper thus combines models from stochastic frontier analysis and spatial econometric literature to assess the level of inefficiency of maternal health care provision in India. The focus on India is relevant because it alone accounts for about a fifth (19%) of total maternal deaths in the world, which means that the target for MDG 5 at a global level cannot be achieved without significant progress in reducing maternal mortality in that country..

The available data was modelled using three variants of stochastic frontier model, including the standard stochastic model, a model corrected for heterogeneity [39] as well as a spatial stochastic frontier model developed and tested in this paper. This confirmed the existence of both inefficiency, heterogeneity and spillover effects in the delivery of maternal care in India. Spill over effects were captured through spatial lag variables with respect to outputs and efficiency distribution.

Consistent with previous work by Greene [31,39] for the case of unaccounted for heterogeneity where the effects show up as inefficiency, the resulting estimates from the spatial stochastic frontier model revealed that the same problem also arises when the model is not corrected for spill over effects. Results showed that the predicted mean efficiency score and the estimated underlying distribution of u (δ_u_) were significantly lower for the spatial stochastic frontier models than those of the classical model and the model with only heterogeneous effects. The range of statistical tests undertaken in the analysis also confirmed that the spatial health frontier models fit the data notably better than both the classical and heterogeneity corrected models. These suggest that in health systems where interactions are a common place failing to correct for spatial externalities may lead to these unaccounted for effects to show up as inefficiency in the analysis. However, this does not necessarily imply that the rankings of spatial units with respect to inefficiency resulting from the spatial stochastic frontier model should be different from that of either the classical or the heterogeneity correct model. But in the data used in the present analysis, the rankings also changed, considerably (see Table 4). In sum, the fact that the spatial lag coefficients are large and significant may suggest the emergence of collective behavior and aggregate patterns in the delivery of maternal care services in the country, which may have been brought about by peer group effects (possibly operating through yardstick competition).

Regarding the role of inputs in the production process, all the three models showed the correct sign and with one exception (untied funding) were all significantly linked to levels of institutional delivery rate. In other words, more health facilities per square kilometres and more qualified health workers per facilities as well as facilities that were well equipped with key amenities lead to significantly high rate of institutional delivery. On the other hand, rresults from the inefficiency analysis showed that most districts in the country were operating well below their maximum capacity. The overall mean inefficiency score in the country was about 38%, while as many as two-third of districts in the country had mean efficiency level of about 75 per cent or less. This suggests that further progress in improving maternal care in India should focus not only on putting new resources but also in ensuring that existing resources are utilised efficiently, especially in parts of the country where inefficiency is extremely low. Finally, we should note that the reported efficiency estimates refer to the efficiency of an output, not the absolute level of the output itself. Thus, in those districts where efficiency level is already high but the rate of institutional coverage is still low further progress can only come by way of putting new resources in these areas. The fact that urbanization, literacy and low proportion of population in lowest income quintile (a proxy measure for income) had an enabling effect on the efficiency of institutional delivery mean that changes in any or all of these can also be expected to improve the system.
